# A novel CD40LG mutation causing X‑linked hyper-IgM syndrome

**DOI:** 10.1016/j.gmg.2024.100007

**Published:** 2024-11-20

**Authors:** Xuejing Li, Yungai Cheng, Dan Xu, Beilei Cheng, Yingchun Xu, Zhimin Chen, Lanfang Tang, Yingshuo Wang

**Affiliations:** Department of Pulmonology, Children’s Hospital of Zhejiang University School of Medicine, National Clinical Research Center for Child Health, Hangzhou 310052, China

**Keywords:** X-HIGM, CD40L, Flow cytometry, Lymphocyte subsets, WES

## Abstract

X-linked hyper-IgM (X-HIGM), which results from mutations of the CD40 ligand gene (CD40LG) located on chromosome Xq26.3, is characterized by a defective T-B lymphocyte cross talk and class switch recombination (CSR). The present study aimed to evaluate the expression of CD40L and lymphocyte subsets using flow cytometry and to identify the novel genetic defect of *CD40LG* responsible for X-HIGM in a Chinese family. We reported an X-HIGM case caused by a novel mutation in CD40LG. The expression of CD40L was absent on the surface of activated CD4 + T cells evaluated using flow cytometry. The total number of mature B cells in circulation was normal, but memory B cells were significantly decreased. In helper T cells, Th2 was dominant, and the numbers of Th1 and Th17 were decreased. The results of genetic analysis revealed a new causative mutation in CD40L (NM_000074;exon5;c.505_506del), which leads to a change in amino acids (p.Y169Lfs*31) appearing in the proband. The frame shift mutation led to incorrect amino acid translation and loss of β-pleated sheet and loop region, which produced a mutant dysfunctional protein. This study provides a complete picture of X-HIGM and broadens our knowledge of the pathogenicity of the CD40L variant spectrum.

## Introduction

Hyper-IgM (HIGM) syndrome is a rare group of primary immunodeficiency disorders (PIDs) characterized by low or absent levels of serum IgG, IgA, and IgE, with normal or elevated serum IgM [1]. As an unusual immunodeficiency disorder, HIGM usually presents in infancy with severe recurrent upper and lower respiratory tract bacterial infections, protracted diarrhea, and opportunistic infections, including *Pneumocystis jirovecii* pneumonia [2]. The morbidity risk of autoimmune disorders, liver disorders, and malignant tumors has been reported to be significantly increased in patients with this disorder [3].

X-linked HIGM (X-HIGM), which results from mutations in the CD40LG located on chromosome Xq26.3, is the most common form of HIGM. CD40LG contains five exons. Although mutations affect the entire CD40LG, the majority are found within exon 5, which comprises most of the tumor necrosis factor homology domain [4]. CD40LG encodes the membrane glycoprotein CD40 ligand (CD40L, CD154, or gp39), which is mainly expressed on the surface of activated CD4 + T cells [5]. CD40L belongs to the TNF family of cytokines, and its binding to CD40 is expressed in B cells, monocytes, and macrophages. CD40L–CD40 binding can promote the stimulation of T cells, boost the differentiation, development, proliferation, and maturation of B cells, and participate in the secretion and class switching recombination (CSR) of immunoglobulin, influencing both T and B cell immunity [6], [7]. To date, more than 190 variants of the CD40L gene have been reported. A large number of gene mutations remain to be identified and explored. A strict relationship between genotype and phenotype has not yet been established in X-HIGM [8], [9].

Here, we report an X-HIGM case caused by a novel mutation in CD40LG whose CD40L expression is absent and whose three-dimensional structure of protein is obviously changed. In this study, we collected the clinical data of a diagnosed X-HIGM child, described his detailed clinical manifestations and laboratory presentations, and then analyzed the expression of CD40L, lymphocyte subsets, and genetics of this case. This provides a complete picture of X-HIGM and broadens our knowledge of the pathogenicity of the CD40L variant spectrum.

## Materials and methods

### Ethical compliance and patient

The study was approved by the Medical Ethics Committee of the Children’s Hospital of Zhejiang University School of Medicine (IRB No.2020-IRB-185) and was performed in accordance with the Declaration of Helsinki. Clinical information about the patient was obtained from the Children’s Hospital of Zhejiang University, School of Medicine. The participants in this study included the patient and his parents. Written informed consent was obtained from all participants.

### Sample collection

Fresh whole venous blood (5 ml) samples were collected from the patient in sodium heparin anti-coagulated syringes and delivered to the laboratory within 4 h. About 2 ml of blood was used for genetic sequencing, and the remainder was used for the isolation of peripheral blood mononuclear cells (PBMCs). Additionally, 4 ml blood samples were collected from the participating family members for genetic analysis and detection of CD40L using flow cytometry.

### Detection of CD40L and lymphocyte subsets using flow cytometry

The expression of CD40L on CD4 + T cells was detected using flow cytometry. PBMCs from all participants were isolated with density gradient centrifugation on a lymphocyte-separating medium. PBMCs were stimulated with ionomycin (500 ng/ml) and phorbol 12-myristate 13-acetate (PMA) (50 ng/ml) for 4 h in a CO_2_ incubator at 37 °C, and non-adherent cells were collected. The activated cells were then incubated for 30 min at 4 °C with phycoerythrin (percp)-conjugated anti-human anti-CD4 and BV510-conjugated anti-human CD154 antibodies. Next, cells were detected using a flow cytometer (FACSCanto II, BD Biosciences) and analyzed with FlowJo software (Tree Star, Ashland, OR, USA). The percentage of CD154 + cells was determined by gating on dot-plot histograms. EDTA blood samples were used, and staining for lymphocyte surface markers was performed after red cell lysis, according to a standard flow cytometric multicolor protocol. Lymphocyte subsets were measured using multicolor flow cytometry panels.

### Genetic analysis and bioinformatics analysis

Genomic DNA was extracted from peripheral whole blood using a QIAamp DNA Mini Kit (Qiagen Inc.). The DNA was interrupted and fragmented at an average size of 180 bp with a Bioruptor sonicator (Diagenode). Then, target gene regions were captured and enriched using the GenCap Medical exon capture kit. Libraries were prepared and sequenced on an Illumina HiSeq X Ten platform. *Whole-exome sequencing (WES)* was used to search for genetic variants. To validate the suspected variant generated from *WES*, the target sites and their flanking sequences were amplified using PCR combined with Sanger sequencing. The genetic mutations were confirmed in the patient’s parents using the same procedure. Potential pathogenic genetic mutations were identified using bioinformatics analysis and analyzed in the HGMD, dbSNP, and Clinvar databases to confirm whether they had been reported previously. The CD40LG gene sequence (NM_000074) was obtained from NCBI (https://www.ncbi.nlm.nih.gov/). The tertiary structure of the CD40LG protein was acquired from the AlphaFold Protein Structure Database (https://alphafold.ebi.ac.uk/) [10]. The three-dimensional structures of CD40LG wild type and mutant type (p.Y169Lfs*31) were generated with homology modeling using the three-dimensional structure visualization software Swiss-PdbViewer.

## Results

### Clinical data

A male infant was admitted to the hospital with a one-month chronic cough and repeated cyanosis for two weeks at the age of four months on July 24, 2019. He was the second child of his parents and was a full-term baby with a birth weight of 3500 g. His mother was diagnosed with a congenital atrial septal defect and was cured with surgery when she was six years old. His parents and other family members had no related symptoms, and there was no history of PID in the maternal family. The parents were non-consanguineous. Two months prior, he had been treated with intravenous antibiotics for eight days due to left axillary cellulitis, and had gradually recovered after receiving incision and drainage at a primary local hospital.

**Fig. 1:**
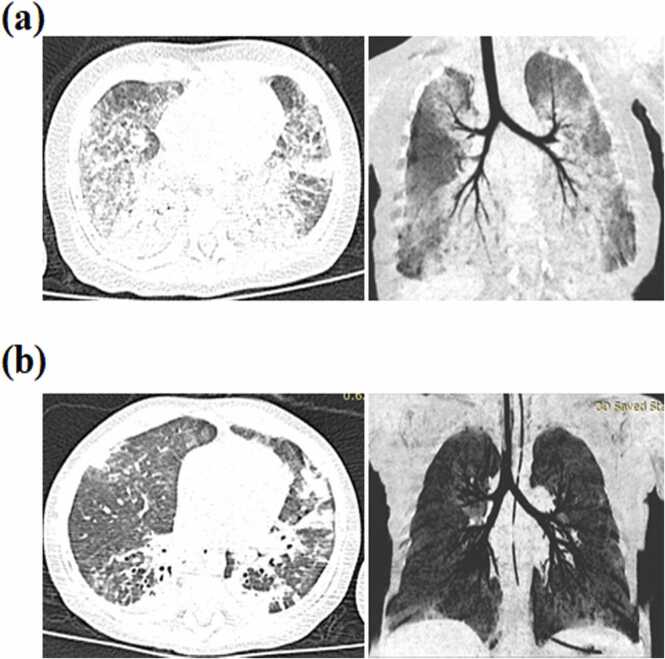
Lung windows from high-resolution CT scans of the patients. (a) Bilateral diffuse exudative and ground-glass opacity changes. (b) The shadow was obviously absorbed after 4 weeks.

On admission, he suffered shortness of breath and moaned constantly in his mother’s arms. Pulse oxymetry showed SpO_2_ of 90 % in room air, which rose to 96 % with oxygen supplied at 2 L/min. His vital signs were as follows: body temperature 36.7 °C, pulse 132/min, respiration 56/min, and blood pressure 102/67 mmHg. Physical examination revealed no rale or wheezing breathing sounds on chest auscultation, despite tachypnea with a triple concave sign. Chest computed tomography (CT) showed bilateral diffuse exudative and ground-glass opacity changes (Fig. 1a). Echocardiography revealed an ejection fraction of 68 % without any anatomical or functional abnormalities. A peripheral blood test revealed a white blood cell count of 12 × 10^9^/L (reference value: 5–12 × 10^9^/L) with an absolute neutrophil count of 7.4 × 10^9^/L (with 67.8 % neutrophils), 140 g/L hemoglobin, platelet of 428 × 10^9^/L, and C-reactive protein levels < 0.5 mg/L. His initial immunological results showed low IgG (0.2 g/L, normal: 3.06–7.74 g/L), low IgA (0.01 g/L, normal: 0.05–0.41 g/L), normal IgM levels (0.74 g/L, normal: 0.24–0.88 g/L), and normal IgE levels (<18.9 g/L, normal: 0–100 g/L). On the second day after admission, his respiratory rate increased to 60–80 breaths/min, and mechanical ventilator care was initiated after he was transferred to the intensive care unit (ICU). We then found *Pneumocystis jirovecii* (*P. jirovecii*) using “next-generation” sequencing technology (NGS) from his bronchial washing fluid on the third day. No evidence of abnormality was found in other laboratory examinations, including electrolyte, liver and kidney function, HIV, Epstein-Barr virus, influenza virus, adenovirus, respiratory syncytial virus, parainfluenza virus, human metapneumo virus, human bocavirus, human rhinovirus, and Mycoplasma pneumonia. Based on the clinical and laboratory findings, the preliminary diagnoses were *Pneumocystis jirovecii* pneumonia (PJP), HIGM, and acute respiratory distress syndrome. He was treated with trimethoprim/sulfamethoxazole (TMP/SMX) to overcome the infection and intravenous immunoglobulin (IVIG) for supportive therapy. His oxygenation improved, and the ventilator was evacuated on the tenth day. Although chest CT showed that the shadow was absorbed after four weeks (Fig. 1b), he was still reliant on oxygen. Finally, he recovered and was discharged from the hospital for intermittent home oxygen therapy.

### Expression of CD40L and lymphocyte subsets

**Fig. 2:**
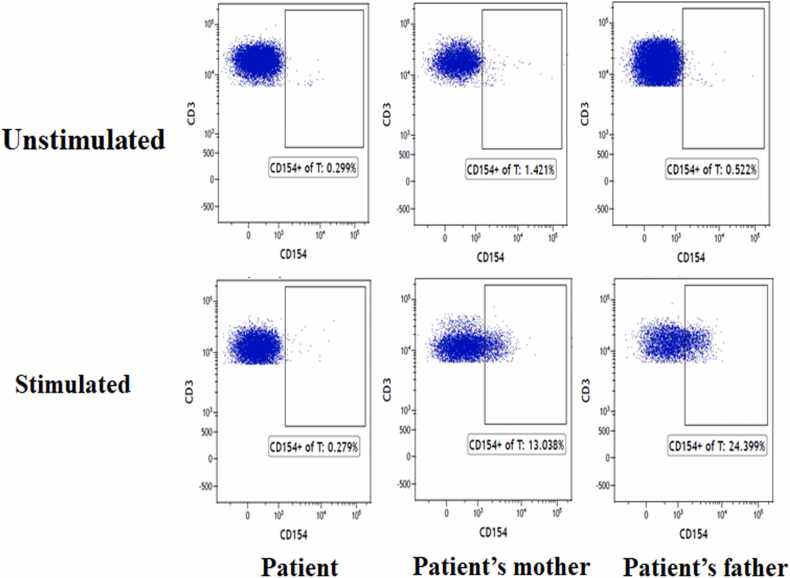
CD40L expression in activated lymphocytes. CD40L expression on the surface of resting (unstimulated) and PMA/ionomycin-activated (stimulated) T lymphocytes from the patient and his parents was analyzed using flow cytometry.

CD40L expression on the surface of resting and PMA/ionomycin-activated CD4 + peripheral blood lymphocytes from the patient and his parents was analyzed using flow cytometry. In the resting state, only a low level of CD40L protein expression was seen on the CD4 + T cells (0.299 %, 1.421 %, and 0.522 %, respectively) (Fig. 2). After in vitro stimulation, his parents showed increased expression (up-regulation) of CD40L on the surface of the CD4 + T cells (13.038 % and 24.399 %), while the patient did not show increased expression of CD40L on activated T cells (0.279 %) (Fig. 2). The patient’s lymphocyte subsets were analyzed at 1 year and 10 months of age. The distribution of the T and B cells and their subsets in the patient is shown in Table 1. There was no abnormality in the absolute number and proportion of total T, CD4 + T, or CD8 + T cells. The total number of B lymphocytes, transitional B cells, and initial B cells were increased, and the memory B cells were significantly decreased. In helper T cells, Th2 was dominant, and the numbers of Th1 and Th17 were decreased. In addition, the patient exhibited a higher percentage of central memory helper T cells (CD4 + TCM) and central memory cytotoxic T cells (CD8 + TCM), as well as a lower percentage of effector memory helper T lymphocytes (CD4 + TEM) and effector memory cytotoxic T lymphocytes (CD8 + TEM).

**Table 1:** Distribution of lymphocyte subsets in the patient.

Submet	Percentage (%)	reference range (%)	Number (number/ul)	Reference range (number/ul)
Total T	51.35	43.7 −80.5	1554.40	711 −2353
CD4 +	69.15	28.06 −70.72	1074.83	368 −1632
CD8 +	26.64	16.37 −42.35	414.10	201 −931
CD4 + TCM	14.42	7.19 −26.33	224.10↑	106.2 −196.9
CD4 + TEM	7.87↓	17.24 −39.12	122.40↓	149.4 −242.1
CD8 + TCM	0.94	0.37 −1.63	14.62↑	4.909 −12.64
CD8 + TEM	2.27↓	8.08 −27.96	35.32↓	50.34 −98.14
Th1	12.07↓	12.65 −36.24	84.86↓	89.88 −140.7
Th2	77.79	22.38 −60.19	804.96	101.7 −738.0
Th17	2.31↓	7.13 −24.53	23.93↓	69.11 −112.8
Total B	30.28↑	5.00 −18.00	987.12↑	90.31 −155.1
Transitional B	10.65↑	0.77 −5.99	105.16↑	1.14 −12.54
Naïve B	75.16	64.21 −87.32	741.95↑	79.27 −175.1
Memory B	2.88↓	8.34 −34.76	8.43↓	15.94 −55.64
NK	6.9	7.00 −40.00	224.86	170.8 −276.5

### Mutation in the patient’s exon 5 of CD40LG

**Fig. 3:**
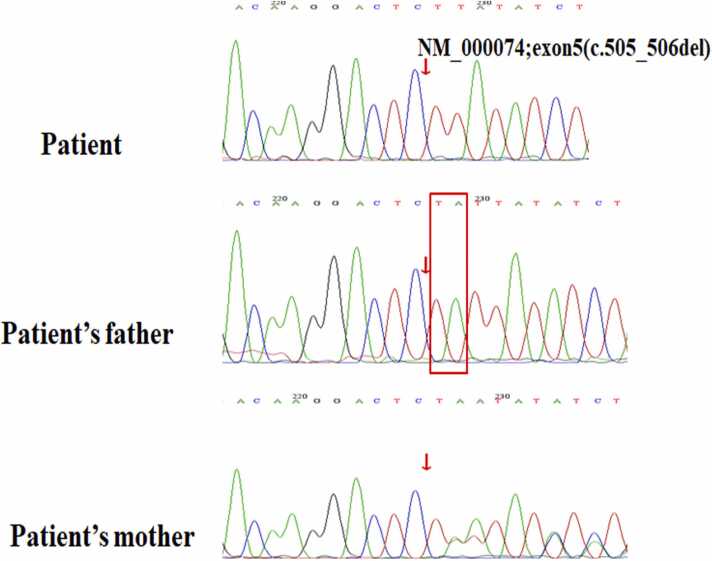
Genetic analysis. Chromatogram showing the patient’s deletion of T and A in exon 5 of CD40LG (c.505_506del; p.Y169Lfs*31) and the heterozygous genotype of the patient’s mother, in contrast to the patient’s father.

WES analysis identified a novel hemizygous mutation on exon 5 of CD40LG (NM_000074;exon5;c.505_506delTA) (Fig. 3), which brought about p.Y169Lfs* 31. The deletion of thymine and adenine in position 505_506 led to a frameshift mutation with a premature stop-codon at amino acid 200 of the CD40L protein. The patient’s mutation was not found in any of the databases that were searched (HGMD, dbSNP, and Clinvar databases), confirming its novelty. Molecular analysis of maternal DNA showed that this locus was a heterozygous mutation, while his father had no variant at the same site.

### Structure prediction of the mutant protein

**Fig. 4:**
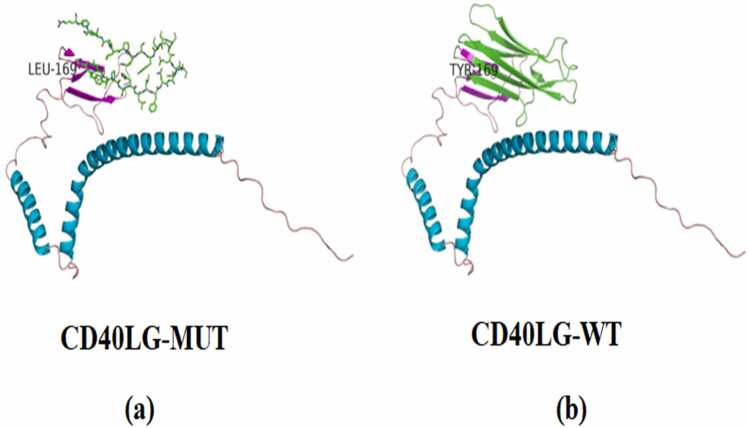
Prediction of the structure of the mutant protein. (a) The mutant protein structure of CD40LG (CD40LG-MUT) was predicted by AlphaFold and Swiss-PdbViewer. (b) Wild-type CD40LG protein structure (CD40LG-WT).

The mutation c.505_506del results in the substitution of leucine for tyrosine at 169 codon, and frame shifting of the following amino acid sequence. Moreover, the structures of protein predicted by the AlphaFold and Swiss-PdbViewer server program showed that the mutant p.Y169Lfs* 31 led to the loss of β-pleated sheet and loop region after the 169th amino acid, which changed the tertiary structure of the protein. (Fig. 4).

### Follow-up and outcome

During the next two years of follow-up, although the patient was prescribed human IVIG replacement therapy at 500 mg/kg/month, he presented with frequent and serious infections, including two episodes of pharyngotonsillitis and seven episodes of pneumonia (five of which needed non-invasive or ventilator-supported treatment in the ICU). Because he needed intermittent oxygen inhalation at home to improve his hypoxia, his parents worried that he could not tolerate hematopoietic stem cell transplantation (HSCT). Although hematopoietic stem cell transplantation was the only curative treatment, they chose not to try. Unfortunately, he died from severe pneumonia at the age of 2.5 on October 30, 2021.

## Discussion

X-HIGM syndrome is a rare, inherited immune deficiency disorder characterized by recurrent infections with low levels of IgG and IgA, and normal or increased levels of IgM. In this study, we reported a new mutation of CD40LG (NM_000074;exon5;c.505_506del) in a four-month-old male child with X-HIGM. The frame shift mutation led to incorrect amino acid translation and loss of β-pleated sheet and loop region, producing a mutant dysfunctional protein. The expression of CD40L, detected with flow cytometry on the surface of activated CD4 + T cells, was absent in the patient. The above results, together with the clinical information, provided strong evidence that this was a novel mutation type of X-HIGM.

The most prominent clinical feature observed in X-HIGM patients is recurrent respiratory infection, and *Pneumocystis jirovecii* is the most common respiratory pathogen [3], [11], [12]. X-HIGM results in a defect in Ig isotype switching and an increased susceptibility to opportunistic infections [13]. *Pneumocystis jirovecii* pneumonia is the first clinical presentation in more than 40 % of infants diagnosed with X-HIGM and is shown as the pathogenic organism in roughly 30 % of individuals with X-HIGM [11], [14], [15]. It accounts for 10–15 % of the mortality associated with X-HIGM [11], [16]. In our study, the boy showed recurrent respiratory infections during infancy and suffered from PJP at four months of age. Severe axillary cellulitis and PJP indicated that the boy may have had an underlying PID, so WES analysis was applied to detect the underlying genetic defects. It is difficult to distinguish *P. jirovecii* infection from respiratory tract infection due to other causes, especially when there is no evidence of an underlying immune deficiency. However, when common viral and bacterial pathogens are not identified, PJP should be suspected in infants with severe interstitial pneumonia accompanying normal breathing sounds, whether or not there is evidence of immune deficiency [17]. In addition, pediatricians should consider that PJP may be the initial clinical symptom of PID [18], [19]. Underlying PID should be evaluated in infants using molecular genetic testing.

X-HIGM results from mutations in CD40LG, which encodes the membrane glycoprotein CD40L. CD40L, also referred to as CD154 or gp39, is a 32-kD protein of 261 amino acids and belongs to the TNFα superfamily, mainly expressed on the cell surface of activated CD4 + T cells [5]. CD40L is a Type II transmembrane protein formed by an intracytoplasmic region, a short transmembrane domain, and an extracellular portion with a TNF-homology domain [20]. As expected, the patient exhibited absent expression of CD40L on activated CD4 + T cells after stimulation. It is of note that the expression of CD40L after stimulation of his mother, who had a heterozygous mutation, was lower than that of his father, who had a normal genotype. However, this low expression of CD40L in the X-HIGM carrier in our study did not cause any clinical symptoms of immune deficiency. This confirms previous research theories that a small population of T cells expressing wild-type CD40L is sufficient to prevent the clinical symptoms of X-HGIM [21], [22]. Typically heterozygous females are asymptomatic, but skewed X-chromosome inactivation could lead to phenotype expression of the X-HIGM in females [21], [23]. Because CD40L expression measures use CD40L binding to detect when the extracellular domain of CD40L (which binds to the CD40 receptor) is normal, but the intracellular signaling pathway from CD40L is nonfunctional [15], [24], we did not find evidence of HIGM through CD40L expression testing. Thus, this testing should not be used as the only diagnostic test when HIGM1 is suspected; genetic testing is required for diagnosis.

CD40–CD40L interaction is an essential signal for B cell proliferation, expression of activation markers, immunoglobulin production, and isotype switching. In addition, CD40–CD40L binding can induce the formation of B memory cells and germinal centers, and signaling through CD40 prevents the apoptosis of germinal center B cells [7], [25]. X-HIGM syndrome causes a limitation in immunoglobulin isotype switching in B cells, resulting from defects in the CD40–CD40L signaling pathway, and therefore, normal or elevated IgM levels and a total absence of other isotypes [7], [26]. Consistent with this theory and previous X-HIGM reports, the patient had low IgG, IgA, and IgE with normal IgM. Furthermore, in our study, the numbers of total B lymphocytes, transitional B cells, and initial B cells were increased, while memory B cells were significantly decreased (Table 1), which indicates that B cells had normal activation function but abnormal maturation and could not secrete normal immunoglobulin. The total number of mature B cells in circulation was normal, but they could not be converted into memory B cells, so the number of class-switched memory B cells was markedly reduced [27], [28].

Memory T cells also provide immunological memory protection for the body. Memory T cells can be divided into two distinct subgroups: central memory T cells (TCM) and effector memory T cells (TEM). TEM mediates protective memory, and it migrates to peripheral inflammatory sites and exerts effector functions. Reactive memory is played by TCM, which has almost no effect function, but can stably proliferate and differentiate into effector cells under the stimulation of antigens [29]. Du *et al.* observed a lower percentage of central memory CD4 + T cells in CD40L-deficient patients [30]. In our study, the patient exhibited a higher percentage of CD4 + TCM and CD8 + TCM, as well as a lower percentage of CD4 + TEM and CD8 + TEM. Therefore, the heterogeneity of TCM and TEM requires further research. In helper T cells, Th2 was dominant, and the numbers of Th1 and Th17 were decreased, suggesting that the antibacterial ability and immune regulation were weakened. Palter *et al.* reported a case of X-HIGM with Leishmania infection, and Th1 cell-mediated response is related to an increased susceptibility to Leishmania species infections and Leishmania clearance [31].

In accordance with the clinical presentation of the patient and laboratory findings, the mutation c.505_506delTA in exon 5 of CD40LG was found, which was identified as a novel deletion mutation. CD40LG is the only gene known to cause X-HIGM, and the Human Gene Mutation Database (www.hgmd.cf.ac.uk) lists close to 190 unique mutations in CD40LG, including deletions, insertions, missense mutations, nonsense mutations, and splice site mutations. CD40LG, located on chromosome Xq26.3, contains four introns and five exons. Mutations can be found throughout the five exons of CD40LG [32], with exon 5 bearing the highest number of mutations [33], [34]. Exon 1 encodes the intracellular and transmembrane regions and a small portion of the extracellular domain, while exons 2–5 encode the rest of the extracellular domain. The mutation found in our patient was a 2-nucleotide deletion in exon 5 of CD40LG, which encodes the extracellular C-terminal TNF-homology (TNFH) domain (amino acids 123–261). This portion will no longer bind to its receptor CD40 if mutated [35]. This frame mutation resulted in p.Y169Lfs* 31, which brought about incorrect amino acid translation and a stop after 30 codons. Furthermore, protein structure prediction with AlphaFold and the Swiss-PdbViewer server program produced a mutant protein with loss of β-pleated sheet and loop region, which obviously changed the tertiary structure and stability of the protein.

Current therapeutic strategies used for patients with X-HIGM include HSCT, immunoglobulin replacement, granulocyte colony-stimulating factor (G-CSF) administration, and antimicrobial prophylaxis for opportunistic infections [36]. Nevertheless, even if the patients undergo IVIg replacement treatment, they may also experience life-threatening infections, just like the patient in this study. HSCT offers the only curative therapeutic option. However, no difference in survival was observed between patients treated with or without HSCT, although measures of activities of daily living favored transplantation [3]. Therefore, there is an urgent need for more feasible and personalized therapies. Targeted gene therapy is a permanent and promising curative therapeutic option for X-HIGM, and could offer a better treatment response on the basis of precise genetic diagnosis [37], [38].

## Ethical approval

This work was approved by the Ethics Committee of Children’s Hospital affiliated to Zhejiang University. All the procedures performed in the study were according to the Declaration of Helsinki.

## CRediT authorship contribution statement

Xuejing Li,Yungai Chen and Dan Xu gathered clinical information from the family, performed literature review, and drafted the manuscript. Zhimin chen, Beilei Chen and Yingchun Xu performed molecular genetic analysis. Lanfang Tang and Yingshuo Wang designed the study. All authors revised the manuscript.

## Conflict of Interest

The authors declare that there are no financial or other conflicts of interest.

## Data Availability

The data that support the findings of this study are available on request from the corresponding author. The data are not publicly available due to privacy or ethical restrictions.
